# Supplementary material for: Realistic scenarios of missing taxa in phylogenetic comparative methods and their effects on model selection and parameter estimation
Source: PeerJ. 2019 Oct 11;7:e7917. doi: 10.7717/peerj.7917 (PMC6791351; doi:10.7717/peerj.7917)
Supplement: Supplemental Information 1 [file peerj-07-7917-s001.docx]

**Comparing the performance of Akaike information criteria versus likelihood ratio tests to discriminate between Brownian Motion and Ornstein-Uhlenbeck models**

In my simulations without missing taxa (nMT), when I simulated data under Brownian Motion and then fitted the simulated trait values to all four models I tested (BM, OU, BMS and OUM), I found that OU was favored over BM by AICc in 12.2% of the simulations (type I error). This is considerably higher than the 5.5% found by Cooper et al. (2015) when comparing BM and OU using likelihood ratio tests (LRT) Therefore, to further explore and compare the performance of AICc versus LRT to discern between these two models, I ran additional analyses using LRT on the results of my nMT simulations under BM.

In this comparison, I was interested only in BM and OU, so first I compared the AICc values of only these two models, excluding BMS and OUM. I found that the type I error rate (OU incorrectly favored over BM) was 16.9%.

To run the LRTs, because I did not record the log-likelihood values of fitted models in my simulation, I first had to back-calculate the log-likelihood of each model based on their AICc score, using the formula:

$$l=k*\left( \frac{n}{n-k-1} \right)-AICc$$

,where k is the number of parameters of each model and n is the number of tips in my simulated trees (always equal to 300). The number of parameters in BM is two (sigma-square and the trait value at the root), and the number of parameters in OU, as implemented in Ouwie, is three. The three OU parameters in Ouwie under the default settings (which I used) are sigma-square, alpha and theta; the trait value at the root is dropped from the model and instead drawn from the stationary distribution of the OU process (Beaulieu et al. 2012).

Next, I ran LRTs using the function *lr.test* in the R package *extRemes*. For each simulated dataset, the LRT tested the null hypothesis that the simpler, and true, BM model is a better explanation of the data than the more complex OU model. The number of degrees of freedom in an LRT equals the difference in the number of parameters of the models being compared (Lewis et al. 2010); in the present case it is 1.


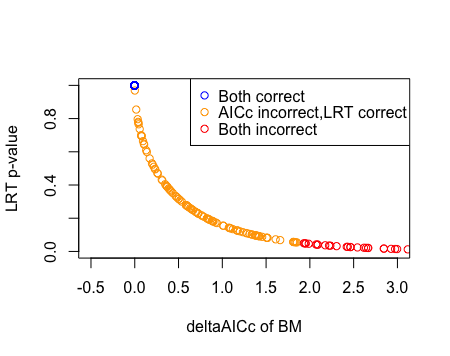
Using a significance threshold of 0.05, I found that LRTs incorrectly selected an OU model over the true BM model in 6.7% of my simulations, a number much lower than the AICc type I error rate of 12.2% and more in line with Cooper et al.’s (2015) 5.5%.

In summary, both AICc and LRT correctly favored BM in 83.0% of the simulations; AICc incorrectly favored OU while LRT correctly favored BM in 10.3% of the simulations; and both approaches incorrectly favored BM in 6.7% of the simulations. In no simulations was AICc correct while LRT was incorrect.

The figure above plots the p-value of the LRTs against the delta AICc of BM for each simulation. A p-value<0.05 means LRT rejects BM, and a delta AICc>0 means AICc rejects BM.

Reference not included in main body of article:

Lewis, F., Butler, A., & Gilbert, L. (2011). A unified approach to model selection using the likelihood ratio test. Methods in Ecology and Evolution, 2(2), 155–162.
